# Alpha 2-adrenoceptor participates in anti-hyperalgesia by regulating metabolic demand

**DOI:** 10.3389/fphar.2024.1359319

**Published:** 2024-03-21

**Authors:** Ke Zhang, Yu-Qing Ren, Yan Xue, Dongxia Duan, Tong Zhou, Ying-Zhuo Ding, Xiang Li, Wan-Kun Gong, Jiao-Qiong Guan, Le Ma

**Affiliations:** ^1^ Department of Anesthesiology, Affiliated Shanghai Sixth People’s Hospital, Shanghai Jiao Tong University School of Medicine, Shanghai, China; ^2^ Shanghai Key Laboratory of Psychotic Disorders, Brain Health Institute, National Center for Mental Disorders, Shanghai Mental Health Center, Shanghai Jiao Tong University School of Medicine, Shanghai, China; ^3^ Department of Anesthesiology, Renji Hospital, Shanghai Jiao Tong University School of Medicine, Shanghai, China; ^4^ Shanghai Eye Disease Prevention and Treatment Center/Shanghai Eye Hospital, Department of Pharmacy, Shanghai, China; ^5^ Department of Orthopedics, The Fifth People’s Hospital of Shanghai, Fudan University, Shanghai, China

**Keywords:** α2-adrenoceptor, pain, transcriptome, cardiovascular-mitochondrial interaction, metabolic processes

## Abstract

The α2-adrenoceptor agonist dexmedetomidine is a commonly used drug for sedatives in clinics and has analgesic effects; however, its mechanism of analgesia in the spine remains unclear. In this study, we systematically used behavioural and transcriptomic sequencing, pharmacological intervention, electrophysiological recording and ultrasound imaging to explore the analgesic effects of the α2-adrenoceptor and its molecular mechanism. Firstly, we found that spinal nerve injury changed the spinal transcriptome expression, and the differential genes were mainly related to calcium signalling and tissue metabolic pathways. In addition, α2-adrenoceptor mRNA expression was significantly upregulated, and α2-adrenoceptor was significantly colocalised with markers, particularly neuronal markers. Intrathecal dexmedetomidine suppressed neuropathic pain and acute inflammatory pain in a dose-dependent manner. The transcriptome results demonstrated that the analgesic effect of dexmedetomidine may be related to the modulation of neuronal metabolism. Weighted gene correlation network analysis indicated that turquoise, brown, yellow and grey modules were the most correlated with dexmedetomidine-induced analgesic effects. Bioinformatics also annotated the involvement of metabolic processes and neural plasticity. A cardiovascular–mitochondrial interaction was found, and ultrasound imaging revealed that injection of dexmedetomidine significantly enhanced spinal cord perfusion in rats with neuropathic pain, which might be regulated by pyruvate dehydrogenase kinase 4 (pdk4), cholesterol 25-hydroxylase (ch25 h) and GTP cyclohydrolase 1 (gch1). Increasing the perfusion doses of dexmedetomidine significantly suppressed the frequency and amplitude of spinal nerve ligation-induced miniature excitatory postsynaptic currents. Overall, dexmedetomidine exerts analgesic effects by restoring neuronal metabolic processes through agonism of the α2-adrenoceptor and subsequently inhibiting changes in synaptic plasticity.

## Introduction

Chronic pain is a complex sensory or emotional experience (Frediani and Bussone, 2019). Nerve injury commonly leads to excessive activation of pain circuits and alterations in spinal-mediated pain transmission and synapse-associated cellular signals (Luo et al., 2014). Inflammatory factors stimulate a variety of calcium ion channels, including calcium store-operated calcium channels and mu-opioid receptors, to promote neuronal firing and pain sensations; specific channel inhibitors or antagonists can significantly inhibit calcium-activated cellular transduction and neurotransmitter release to exert a powerful analgesic role (Ma et al., 2021; Wang et al., 2023). Therefore, finding new intervention targets plays an important role in the development of analgesic drugs.

Synapses consume ATP (Adenosine 5′-triphosphate) to participate in neural plasticity, and the disturbance of energy haemostasis prevents them from performing host functions properly, leading to defective neurotransmission and synaptic connections. Glutamate transmission in the spinal cord is significantly enhanced after nerve injury, and the strength of its synaptic connections is increased by three to five times (Zhang et al., 2019). Chronic pain significantly changes mitochondria-related markers; in particular, it leads to significant damage to mitochondrial DRP1 and persistent overactivation of ROS (reactive oxygen species) (Dai et al., 2020). Macrophages actively control the expansion of an inflammatory damage away from the inflammation site by transferring mitochondria to sensory neurons (van der Vlist et al., 2022). During the regression of inflammatory pain in mice, M2-like macrophages infiltrate the dorsal root ganglion containing the cellular body of sensory neurons and resume the oxidative phosphorylation of these neurons (Niehaus et al., 2021; van der Vlist et al., 2022). Urolithin A improves mitochondrial autophagy and respiration in primary chondrocytes during the pathological state of arthritis, thereby reducing cartilage degeneration, synovial inflammation and pain; such improvements are associated with increased mitochondrial autophagy and mitochondrial content in the joints of mice (D Amico et al., 2022). A mutual response exists between mitochondria and local blood vessels, and it guides the supply of substances to maintain mitochondrial respiration and ATP production (Marcu et al., 2017). Transient pain signal transduction requires enhanced blood flow to satisfy the synapse demand (Claron et al., 2021). Therefore, the imbalance of energy metabolism may be the key cause of pain.

The α2-adrenoceptor agonist dexmedetomidine (DEX) is a commonly used sedative in clinical practice and has analgesic effects; however, its mechanism of analgesia in the spine remains unclear. In this study, we investigated the effects and molecular mechanisms of DEX in pain by transcriptome sequencing, behavioural, electrophysiological and ultrasound imaging. Our results show that spinal nerve injury leads to changes in spinal transcriptome expression, and the differential genes are mainly related to calcium signalling and tissue metabolic pathways. DEX modulates mitochondrial activity. The mRNA expression of α2-adrenoceptor was significantly upregulated, and it was significantly co-localised with markers mainly in neuronal markers. Intrathecal DEX suppressed neuropathic pain and acute inflammatory pain in a dose-dependent manner. The transcriptome results also demonstrated that the analgesic effect of DEX may be related to the modulation of neuronal metabolism. A cardiovascular-mitochondrial interaction exists, and ultrasound imaging showed that injection of DEX significantly enhanced spinal cord perfusion in rats with neuropathic pain. Increasing the perfusion doses of DEX significantly suppressed the frequency and amplitude of spinal nerve ligation-induced miniature excitatory postsynaptic currents (mEPSCs). Dexmedetomidine exerts analgesic effects by restoring neuronal metabolic processes through agonism of the α2-adrenoceptor and subsequently inhibiting changes in synaptic plasticity.

## Methods

### Rat model of chronic pain induced by spinal nerve ligation

Two-month-old adult male Wistar rats were intraperitoneally injected with 50 mg/kg pentobarbital sodium. After anaesthesia, the rats were opened along the midline of the iliac bone in the back with a length of about 2 cm. The left muscle was bluntly separated. Two parallel spinal nerves L4 and L5 were observed. Relative to the L4 nerve, the spinal L5 was located close to the vertebrae. The L5 nerve was gently stirred and ligated with a hook made of glass electrodes. A medical–surgical blade was used to remove the fascia near the sacrum, detect the relatively vertical L6 nerve, lates it, uses an appropriate amount of penicillin to prevent bacterial infection and closes the wound. The sham operation group was similar to the spinal nerve ligation group, where spinal nerves L5 and L6 were separated but only medical surgical lines were used to surround these nerves without ligation. At 10 days after the operation, the adaptive environment and handle rats were started. The mechanical pain threshold of the left hind foot of the rats was measured by Von Frey’s electronic pain meter. Rats that weighed less than 8 g and had no significant difference in movement posture on both sides were regarded as successful modelling and used for subsequent experiments (Deuis et al., 2017).

### Catheterisation of subarachnoid space in rats

The experimental rats were anesthetised by intraperitoneal injection of pentobarbital sodium (50 mg/kg). The iliac crest position was marked. A 22 cm polyethylene catheter (PE-10 catheter: inner diameter: 0.30 mm; external diameter: 0.55 mm) was implanted into the arachnoid space of the spine, and a typical tail flashing phenomenon was observed. The tip of the catheter was close to the vertical position of the last rib and spine of the rat, and the catheter was fixed at the neck and iliac bone. The catheter was left from the skin of the neck of the rat to prevent the animal from biting and damaging it. An appropriate amount of penicillin was administered to the skin opening to prevent surgical infection and close the wound. The rats were transferred to a heated blanket to restore consciousness and then placed in a single cage. Two days after recovery, the intubation was checked, and the rats were injected with 10% lidocaine in artificial cerebrospinal fluid through the neck catheter by using a 15 μL micro-sample injector, followed by a 15 μL artificial cerebrospinal fluid flush tube. The rats had bilateral posterior foot paralysis and weakness, and the bilateral posterior foot movement was the same after the metabolism of anaesthetic drugs. The results indicated the successful placement of intrathecal catheter in rats, and the model was used in subsequent experiments.

### Measurement of pain threshold in rats

The rats were acclimatized to the experimental environment 3 days before the start of the behavioural study. According to the experimental plan, the rats were placed in the laboratory for at least 30 min at the same time every day. On the first day, the rats were placed in a feeding box and were gently stroked on the back for 1 min twice. On the second day, the rats were gently placed on an iron rack and gently stroked along the direction of the hair for 1–2 min each time. Similar operations were performed until the day before the experiment. This operation allows the rats to be familiar with the experimental operator through odor and steps conducted, reduce the adverse emotional changes caused by the operation by establishing a familiar relationship between the operator and the tested rats and reduce errors and inaccurate measurements that may be introduced by other factors. After handling, the rats were transferred to a clear Plexiglass box to detect the mechanical pain threshold. The box was placed on a metal mesh 40 cm away from the experimental table. After 30 min of adaptation, the rats were stimulated vertically upward at the intersection of the third and fourth toes of the left hind foot by using 2450 CE electronic Von Frey (2450 CE electronic Von Frey) equipped with 15 fibres until the rats showed typical foot retraction or foot lift response when escaping injury. Paw withdrawal latency (mechanical pain threshold) was recorded when the rat avoided injury. The threshold value of each rat at a certain point in time was the mean value of three consecutive measurements with an interval of 3 min.

### Real-time fluorescence quantitative PCR

The rats were injected intraperitoneally with pentobarbital sodium (50 mg/kg). The spine was cut along the iliac bone, and the tissue was separated from the left and right sides with fine surgical scissors to the last rib to quickly separate the spinal cord L3–L5 enlargement. Pre-cooled artificial cerebrospinal fluid was used to clean the residual blood in the enlarged part of the waist. The surgical site was separated by a surgical blade and transferred to liquid nitrogen for rapid cooling and storage at −80°C. Total RNA was extracted by Trizol reagent. All consumables required for the whole experiment were subjected to DEPC (Diethyl pyrocarbonate) water treatment, and the experimental table was treated with RNA enzyme scavenger. Prior to extraction, the preserved tissue was recovered at −80 °C for 5 min at room temperature. About 1 mL of Trizol and three RNA-free homogenising magnetic beads were added to the homogenate at 60 Hz/90 s and split at room temperature for 10 min. The sample was then added with 200 μL of trichloromethane, swirled for 3–5 s and centrifuged at 12,000 rpm/4°C for 10 min. The centrifuged EP tube was then divided into three layers.

The uppermost layer dissolves RNA, and the middle layer dissolves DNA. The middle layer of liquid should not be touched when absorbing the supernatant to prevent mixing into RNA and affecting the reliability of the experiment. The supernatant was added with pre-cooled isopropyl alcohol (1:1), reversed and mixed several times. The sample was placed at −20°C for 2 h and centrifuged at 12,000 rpm/4°C for 10 min. The EP tube was gently reversed to discard the supernatant, and the white precipitate formed was regarded as RNA. The precipitate was added with 1 mL of pre-cooled DEPC water with 75% ethanol, reversed several times, centrifuged at 7,500 rpm/4°C for 10 min and washed repeatedly. After discarding the supernatant, a 10 µL tip head was used to remove excess liquid from the tube wall. The sample was left at room temperature until the RNA dried. The RNA was then dissolved in 50 µL of DEPC water. RNA concentration and purity was measured by Nanodrop, and the qualified RNA A260/A280 was between 1.8 and 2.0, which can be used for further experiments.

Here are the primers for the experiment:

Ch25 h: Forward primer 5′- CCT​AAG​TCA​CGT​CCT​GAT​CTG​C-3′,

Reverse primer 5′- GAG​GAC​GAG​TTC​TGG​TGA​TGC​A-3’;

Itpkc: Forward primer 5′- TAC​CAG​CGA​TCC​TGA​GGA​CAG​A-3′,

Reverse primer 5′- AGT​GCT​TAT​GGA​AGG​AGA​CCA​CG-3’;

Gch1: Forward primer 5′- AGC​AAG​TCC​TTG​GTC​TCA​GTA​AAC-3′,

Reverse primer 5′- ACC​GCA​ATC​TGT​TTG​GTG​AGG​C-3’;

Chsy1: Forward primer 5′- CGA​CAG​GAA​CTT​TCT​CTT​CGT​GG-3′,

Reverse primer 5′- CCT​CGC​TAG​AGA​AGA​ACT​CCA​C-3’;

Arl5c: Forward primer 5′- GAG​GAG​CTG​TAT​AAG​ATG​CTG​GC-3′,

Reverse primer 5′- GGT​GGT​CTT​TGA​TGG​CAC​TGA​G-3′

Pdk4: Forward primer 5′- GTC​GAG​CAT​CAA​GAA​AAC​CGT​CC-3′,

Reverse primer 5′- GCG​GTC​AGT​AAT​CCT​CAG​AGG​A-3′

GAPDH: Forward primer 5′-ACC​ACA​GTC​CAT​GCC​ATC​AC-3′,

Reverse primer 5′- TCC​ACC​ACC​CTG​TTG​CTG​TA-3′

### Immunofluorescence staining

Tissue separation: The rats were anesthetised by intraperitoneal injection of pentobarbital sodium (50 mg/kg). The thorax was cut along the sternal angle, the apex of the heart was exposed and the right auricle was cut quickly. About 100 mL of pre-cooled normal saline was injected to empty the circulating blood. The liver tissue slowly turned white from being red. About 100 mL of 4% paraformaldehyde solution was continuously injected. The tissue was then separated along the iliac bone towards the neck, the enlarged lumbar part of the spinal cord L3-L5 was removed and the outer membrane of the tissue was gently separated in normal saline.

Fixation and dehydration: The removed tissue was immediately transferred to 4% paraformaldehyde solution, stored overnight at 4°C and then transferred to 30% sucrose solution prepared by PBS until the tissue completely sank to the bottom of the solution. The solution was replaced with a new one to completely sink to the bottom to complete tissue dehydration. The dehydrated frozen section tissue was washed twice in phosphate buffer saline (PBS) and placed on clean dust-free paper to completely absorb moisture. The tissue was marked on the opposite side of the blade and transferred to an embedding box with an OTC embedding agent. After forming at −80°C, the sample was transferred from container glass to sample holder for fixation. The sample was sliced with Leica frozen microtome. The ambient temperature of the microtome box was −20°C, and the temperature of the cutter head was −20°C. The spinal cord tissue layer V was sliced in the direction of the cutter head with a thickness of 30 μm. The slices were placed on a 12-well plate, immersed in PBS and stored at 4°C for 1 week.

Blocking: The slices were removed with a glass hook and placed into a 2 mL centrifuge tube with a rounded bottom containing 5% BSA/0.5% Triton sealing solution. The sample was incubated at room temperature for 1 h and rotate at 40 rpm in a shaker.

Incubation of primary antibody: Liquid in the centrifuge tube was slowly poured into a 0.22 μm filter. The sections were transferred to six-well plates, wash with PBS for 5 min and transfer to 1%BSA antibody diluting solution. The corresponding antibody was incubated with the primary antibody.

Secondary antibody incubation: The sections were transferred to a 1% BSA-configured antibody diluter, and all fluorescent secondary antibodies were diluted at 1:1000. The sample was incubated at room temperature for 2 h and rotated at 40 rpm. Imaging and fluorescence intensity analyses were performed with Olympus FV10-ASW 4.2 viewer.

### Spinal cord transcriptome sequencing and analysis

The total RNA of spinal cord L3-L5 was extracted using RNeasy Mini Kit (Qiagen), and the A260/A280 values of qualified RNA were between 1.8 and 2.0, which could be used for further experiments. After fragmentation, PCR, purification and other operations were used to build a database for subsequent sequencing. Lian Chuan Biotechnology Co., Ltd. was commissioned to use the second-generation sequencing platform Illumine Novaseq 6000 for sequencing using the PE150 standard. The expression value was normalised to fragments per kilobase of exon model per million mapped fragments (FPKM). Count unit and value were selected for subsequent analysis. In this study, R (Windows 10 64-bit) was used for analysis. A domestic image site was selected and the corresponding configurations were performed after the installation. The required R packages were then set up through the installation. During the transcriptome analysis, differential genes were selected for DESeq2 package analysis, in which genes with log2 (expression change multiple) > log2 (1.5) and corrected *p*-value < 0.05 were included. Signal pathway enrichment was annotated using the Kyoto Encyclopaedia of Genes and Genomes (KEGG) database. The results were presented in the pattern of pathway enrichment and pathway connection relationships.

Weighted gene co-expression network analysis (WGCNA) can use known gene expression information to correlate with the corresponding indicators for significance association analysis according to previous study (DOI:10.1155/2021/9923537). The gene expression is more in line with the scale-free network when taken to the NTH power. WGCNA mainly focuses on the gene association of some key nodes and introduces the concepts of weight network and soft threshold: lognd = k∝-logk β. In general, correlation R^2^ > 0.8 can meet the scaling data distribution, where *d* is the connectivity of nodes, and *k* is a natural number. Based on the screening principle of the soft threshold, the constructed network should be more in line with the scale-free network characteristics. We set the soft threshold values of 0.95, 0.90, 0.85, 0.80, 0.75 and 0.70. The module with β = 8 was selected according to the above parameters for analysis. After the correlation network was constructed, the cluster tree and corresponding modules were visualised. The correlation between the module and gene expression was established, and the gene expression data corresponding to the module was further extracted. The average of gene significance of all genes in a module was defined as modular significance. The modular significance values were compared among different modules. Modules of interest that are highly associated with a particular clinical feature of a disease were selected for subsequent analysis. Genes that achieved highly similar expression relationships were grouped and divided into different modules. Each module was identified and correlated with behavioural phenotypes, such as ligation, nerve damage, pain, anxiety and paw withdrawal threshold (PWT) and included in WGCNA.

### Electrophysiological section of the spinal cord (L3–L5)

The experimental rats were rapidly anesthetised with isoflurane, the back skin was separated from the iliac crest to the last rib and the spine L3–L5 was quickly cut with scissors along the rib perpendicular to the spine and 1.5 cm away from the iliac crest. The spine was transferred to a pre-cooled slicing solution (234 mM sucrose, 3.6 mM KCl, 1.2 mM MgCl_2_, 1.2 mM NaH_2_PO_4_, 12 mM glucose, 2.5 mM CaCl_2_ and 25 mM NaHCO_3_). After 90 s, the separated spine was slowly and gently pushed out the section fluid from a small position in the back end. A 20 mL syringe was used to flush out the spinal cord L3–L5 part with spinal nerve roots. A 1 cm^3^ agar block (1.5%) served as a spinal cord support and was fixed to the sample holder by using 401 glue to prevent it from falling off. Leica VT1200S microtome was used to slice the spinal cord with an amplitude of 1.00 mm, a velocity of 0.06–0.14 mm and a thickness of 300–400 µm. Cerebrospinal fluid (125 mM NaCl, 3 mM KCl, 1.25 mMNaH_2_PO_4_, 26 mM NaHCO_3_, 1 mM MgCl_2_ and 2 mM CaCl_2_) was transferred to oxygen-saturated artificial cerebrospinal fluid at 32°C. Glucose and 10 mM D-glucose were incubated for 30 min at room temperature and restored for 30 min before recording.

### Electrophysiological recording

Lamina II neurons in the dorsal horn of the spinal cord were recorded with a glass electrode made of borosilicate (impedance 4–6 MΩ, diameter 0.5 mm, diameter 1.0 mm). mEPSC was recorded by adding a glass electrode to K^+^ internal fluid (140 mM K-gluconate, 10 mM HEPES, 1.1 mM EGTA, 2 mM MgCl_2_, 2 mm); 3 mM MgATP and 0.3 mM Tris-guanosine triphosphate (adjusted to pH 7.4 by using CsOH), 10 µmol strychnine; 100 µmol picrotoxin (PTX); and 1 µmol tetrodotoxin (TTX) under a clamping voltage of −70 mV. mIPSC was recorded by adding a glass electrode to the CsCI internal solution (140 mM CsCl, 10 mM HEPES, 1.1 mM EGTA, 2 mM MgCl_2_, 2 mm); 3 mM MgATP and 0.3 mM Tris-guanosine triphosphate (adjusted to pH 7.4 by using CsOH); 20 µmol CNQX; 50 µmol D-AP5; and 1 µmol tetrodotoxin in the external solution at a clamping voltage of −70 mV.

### Laminectomy and functional spinal ultrasound imaging (fUS)

Laminectomy was performed between the 12th thoracic and second lumbar vertebrae by intraperitoneal injection of metomidine (0.4 mg/kg) and ketamine (40 mg/kg) under deep anaesthesia. This window allows the entire ultrasonic probe (14 mm) to be positioned in the sagittal plane. Once the laminectomy was complete, the animal was placed on a ‘spinal cord’ stereotaxic frame, in which the lumbar spine was suspended. Lidocaine (Xylovet, Ceva, France, 2 mg/mL) was applied topically to the exposed muscle and to the insertion point of the spinal brace. Anaesthesia was maintained but reduced with subcutaneous infusion by using an injection pump at rates of 0.1 mg/kg/h for metomidine and 12.5 mg/kg/h for ketamine. A waiting time of about 1 h and 30 min after induction was maintained to obtain repeatable results. Imaging was then conducted to obtain a stable and light level of anaesthesia (breathing rate of about 120 bpm). During surgical procedures and imaging, the body temperature of the animals was maintained at 37°C by using heating blankets and intra-rectal probes (Physitemp, Clifton, NJ). Heart and respiratory rates were monitored (Mouse Ox Plus, Ugo Basile, Italy). Each imaging session lasted for 4–6 h. About 2 mL of saline was gently dripped onto the spinal cord (the dura remains intact), and the cavity formed by the laminectomy was filled with an ultrasound gel. The ultrasonic probe (f5-15MHz, 128 components, Vermon, Tours, France) was fixed above the window by using a three-axis motor system. The probe was connected to an ultrasonic ultrafast scanner (Vera Sonics, Kirk-land, WA:128 channels, 62.5 mHz sampling). Data were processed by Neuroscan real-time acquisition software (Iconeus, Paris, France; Inserm Biomedical Ultrasound Technology Research Accelerator, France).

### Materials

The antibodies used in our study. The NeuN Mouse mAb (Millipore, MAB377, 1:1000-1:200); The GFAP Mouse mAb (Millipore, MAB3402, 1:1000-1:200), and the Iba-1 Rabbit mAb (Abcam, ab178846,1:2000-1:500).

### Statistical analysis

All data were calculated using GraphPad Prism 8 software and presented as mean ± standard error of the mean (mean ± SEM). Unpaired student’s t-test and one-way ANOVA were used for statistical analysis. *p* < 0.05 and *p* < 0.01 indicated statistical significance. Data were analysed by experimenters blinded to the experimental conditions of the study.

## Results

### Chronic pain causes abnormal metabolic changes at the lesion site

**FIGURE 1:**
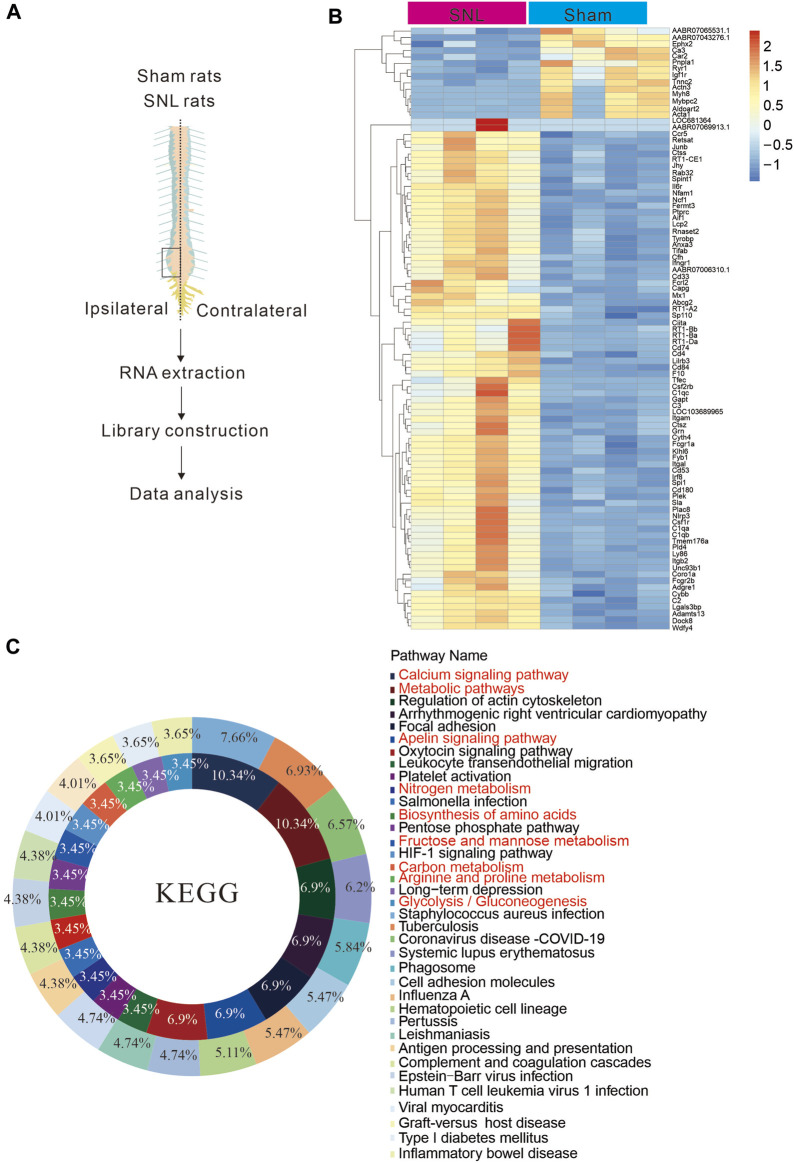
Chronic pain is associated with metabolic abnormalities **(A)** Sequence diagram of spinal nerve ligation chronic pain tissue in rats. **(B, C)**. Tissue sequencing heat map and KEEG analysis results. The results suggest that chronic pain may cause abnormal energy metabo.

Energy metabolism disorders are related to nerve conduction activities and important in neurological diseases. To explore changes in energy metabolism in chronic pain, we used spinal nerve ligation to construct a paradigm in a chronic pain model of rats (Figure 1A). The spinal cords of mice in the spinal nerve ligation (SNL) and sham groups were separated, and the lymphocele site was collected to extract RNA for transcriptome sequencing. During the testing of almost 23,670 genes, differential expression analysis showed enriched genes involved in major cellular processes including calcium signalling pathway, metabolic pathways, apelin signalling, nitrogen metabolism and biosynthesis following neuropathic pain (Figures 1B,C). The results indicated that the signal pathways of glucose, lipids, nutrients and energy metabolism in the spinal cord of rats were significantly changed after chronic pain. Hence, metabolic abnormalities would occur at the pain site, suggesting that metabolic imbalance may be the key cause of pain.

### α-2 Acetylcholine receptor agonist significantly alleviates pain hypersensitivity in rats

**FIGURE 2:**
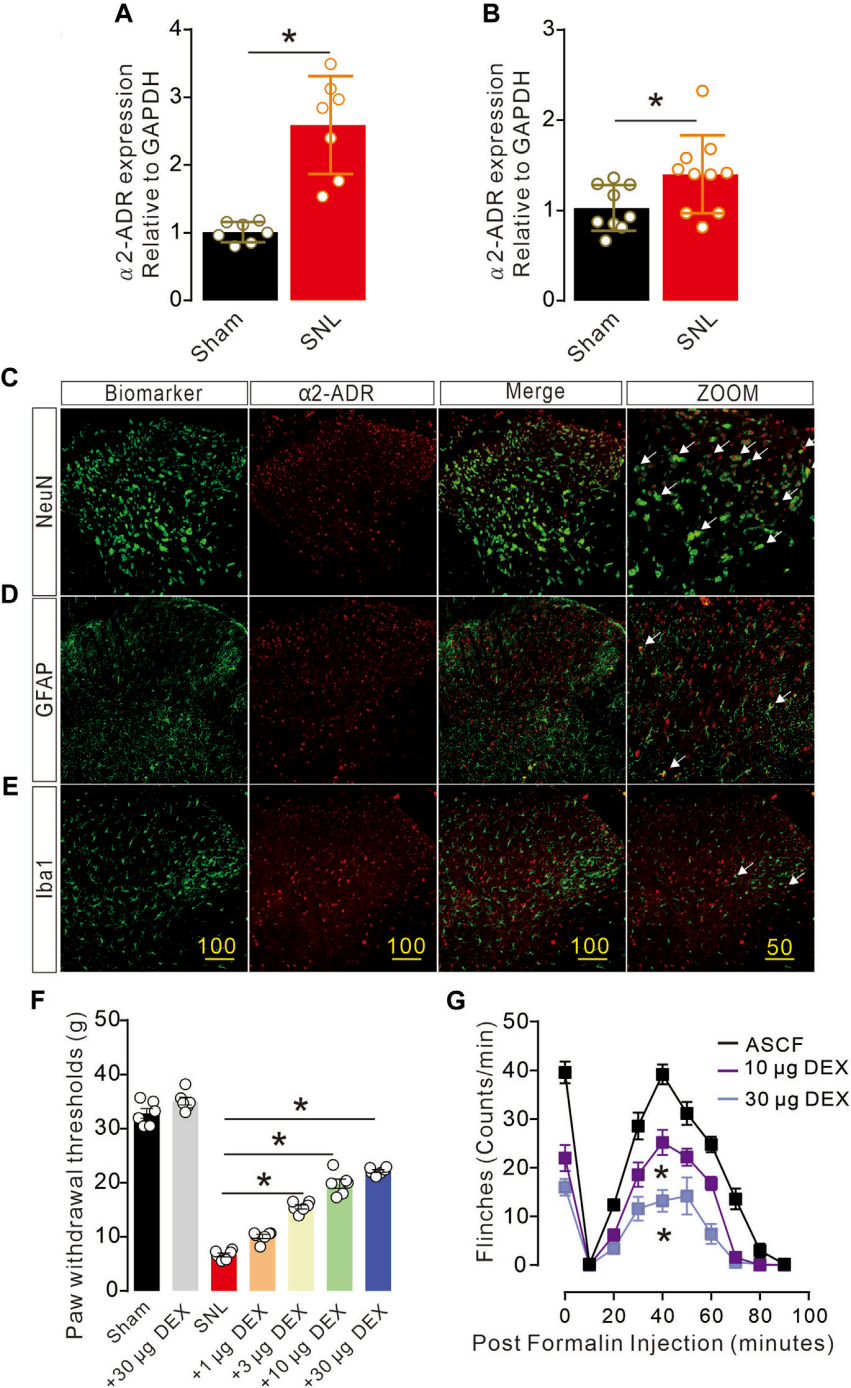
Activation of α2-ADR can improve the pain phenotype of rats **(A, B)**. The QPCR of α2-ADR receptors showed that pain caused a significant increase in presynaptic and postsynaptic α2-ADR receptors (presynaptic: t_12_ = 5.663, *p* = 0.001, two-tailed unpaired t-test; postsynaptic α2-ADR receptors t_17_ = 2.269, *p* = 0.0366 *p < 0.05, **p < 0.01). **(C–F)**. Immunofluorescence staining of α2-ADR receptor distribution showed that α2-ADR receptor was mainly distributed on neurons, and only a small part was distributed on astrocytes and microglia. f. The α2-ADR receptors agonist DEX significantly increased the foot contraction threshold in a dose-dependent manner (F _(6, 35)_ = 311.8, *p* < 0.0001, two-way ANOVA followed by Sidak’s post-tests, *p < 0.05). g. The alpha2-ADR receptor agonist DEX also alleviated formalin-induced inflammatory pain (F _(2, 120)_ = 131.8, *p* < 0.0001, two-way ANOVA followed by Sidak’s post-tests, *p < 0.05).

α2-Acetylcholine receptors (α2-ADR) are metabolically related and involved in pain, but their specific distribution and location in cells and synapses remain unclear. Firstly, we detected the expression of α2-ADR on the presynaptic and postsynaptic membranes with QPCR. The expression of α2-ADR on the presynaptic and postsynaptic membranes was significantly enhanced after the onset of pain (Figure 2A, B). Double immunofluorescence analysis of α2-ADR with NeuN, GFAP and Iba1 was conducted to assess the cell types of α2-ADR. α2-ADR was mainly distributed in neurons, rather than microglia or astrocytes (Figures 2C–E). In addition, the intrathecal agonist DEX significantly suppressed nerve injury-induced neuropathic pain and formalin-induced acute pain in dose gradient-dependent manner (Figures 2F, G).

### Activation of alpha-2 acetylcholine receptors significantly reverses pain-induced changes in metabolic pathways

**FIGURE 3:**
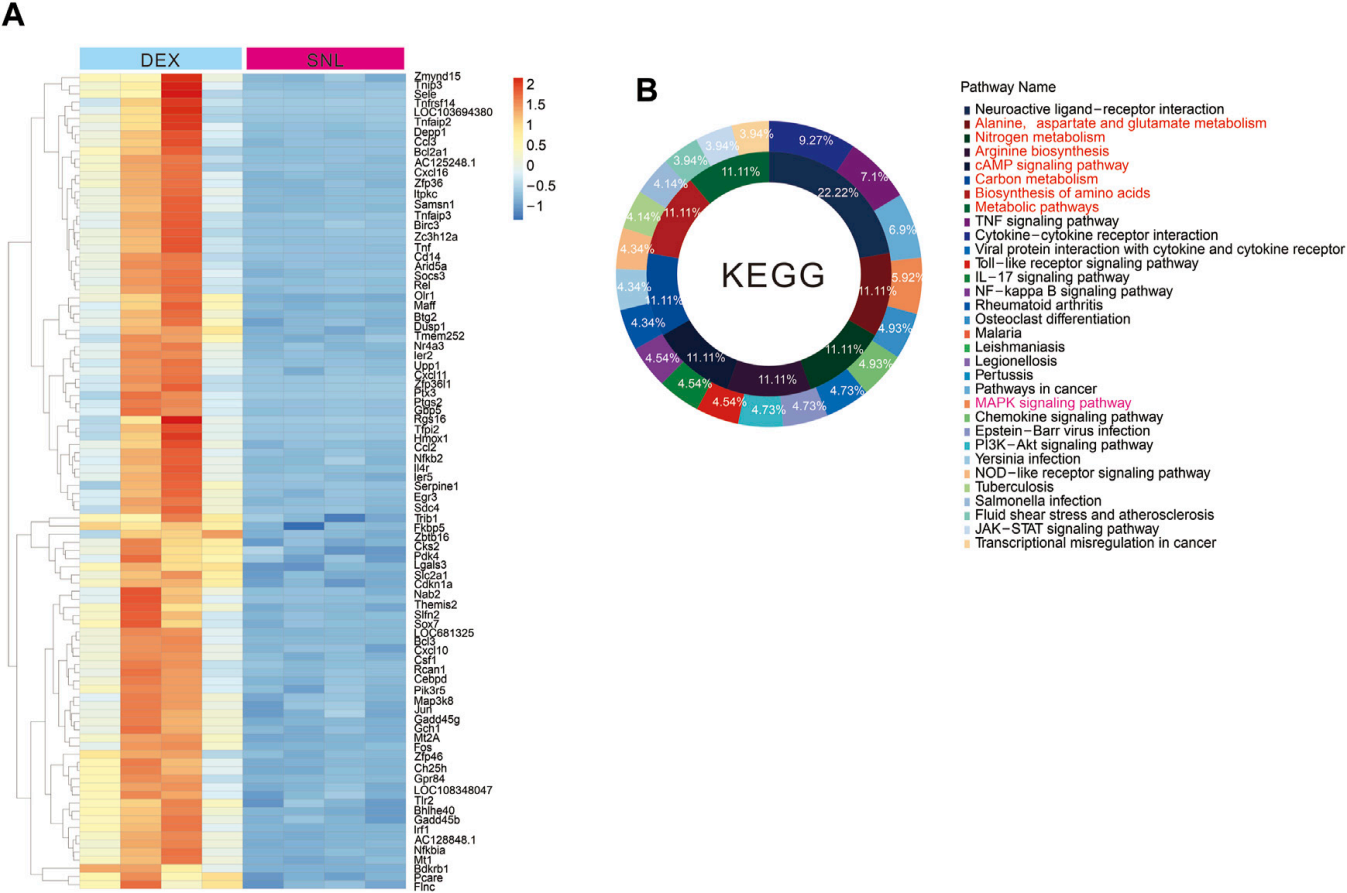
Activation of alpha-2 acetylcholine receptors can improve the metabolic abnormalities caused by pain **(A, B)**. Tissue sequencing heat map and KEEG analysis results. The administration of DEX can significantly change the metabolic abnormalities caused by SNL.

Pain can cause changes in spinal cord metabolic pathways, and the activation of alpha-2 acetylcholine receptors can significantly improve pain phenotypes. By tissue sequencing of SNL and SNL-DEX, we found that administration of the α-2 acetylcholine receptor agonist DEX significantly improved pain-induced metabolic abnormalities, including the following: alanine, aspartate and glutamate metabolism; nitrogen metabolism; arginine biosynthesis; cAMP signalling pathway; carbon metabolism; and biosynthesis of amino acids and metabolic pathways (Figure 3A, B). Therefore, DEX exerted analgesic effects by promoting neuronal metabolism in neuropathic pain.

### WGCNA selects the core module of DEX-induced analgesia

Alpha-2 acetylcholine receptor agonists induced large-scale gene transcriptional alterations. As such, WGCNA was applied to classify and analyse correlated genes and gene expression relationship. The two parts of gene expression cluster analysis and biometric correlation can be linked together. Characteristics associated with neuropathic pain, including spinal nerve ligation, pain, anxiety and PWT (paw withdrawal threshold), can be translated into a data distribution. The detected genes were then categorised according to their similarity principle, and a similar class of genes is grouped into the same module. WGCNAs and gene modules were established based on the expression data of rat spinal cord (L3–L5) samples. Correlation analysis was conducted with the basic characteristics of the samples (L5/L6 spinal nerve ligation, pain, anxiety and foot withdrawal reaction time, etc.) to obtain the key modules of DEX-induced analgesia and related gene pathways.

**FIGURE 4:**
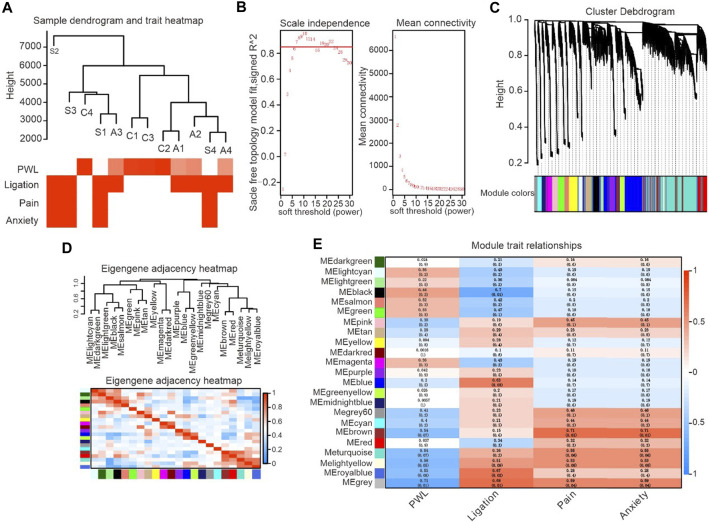
Identification of critical modules correlated with the paw withdrawal latency in DEX-induced analgesic effects. **(A)**. Gene expression system tree map and biometric heat map, representing gene expression and biometric clustering display. **(B)**. WGCNA soft threshold filtering and testing, representing the relationship between Y-axis unscaled fitting data and X-axis soft threshold parameters. **(C)**. The tree map of clustering system was obtained from the database, and the hierarchical linkage clustering method was used. The lower color showed the relationship of gene expression heterogeneity. **(D)**. The figure shows the correlation analysis between the enriched modules and PWL, with red indicating positive correlation and blue indicating negative correlation. **(E)**. The color in the figure represents the correlation coefficient between biometrics and modules, with red representing positive correlation and green representing negative correlation.

After the sample information was corrected and standardised, it was associated with the sample biological indicators. A sample cluster tree and a heat map of biological phenotypes were obtained (Figure 4A, B). To make the data close to the distribution characteristics of scale-free networks, this study determined that the minimum integer value when the fitting coefficient R^2^ approached or reached 0.85 met the analysis requirements. The soft threshold β = 8 also met the data analysis requirements.

WGCNA includes gene sets classified according to similar expression and divides the embryonic gene expression modules through Cluster Tree Cut. Repeated checks were carried out until the data set and the internal structure of the data became stable, and the clustering system tree and corresponding module graph were obtained (Figure 4C). Topological overlap matrix (TOM) was used to describe dissimilarity between data modules. In particular, 1-TOM was used to represent the dissimilarity of gene modules (a value equal to 0 indicates a lack of data connection between two modules) to describe the relationship between modules accurately. Top correlated modules including turquoise, brown, yellow and grey were selected for analysis (correlation coefficient is ranging from 0.71 to 0.54) based on the completed system cluster tree and module heat map to fully understand gene signalling pathways associated with alpha-2 acetylcholine receptor agonism (Figure 4C–E).

### Enrichment analysis of pain-related gene and signal pathway analysis

**FIGURE 5:**
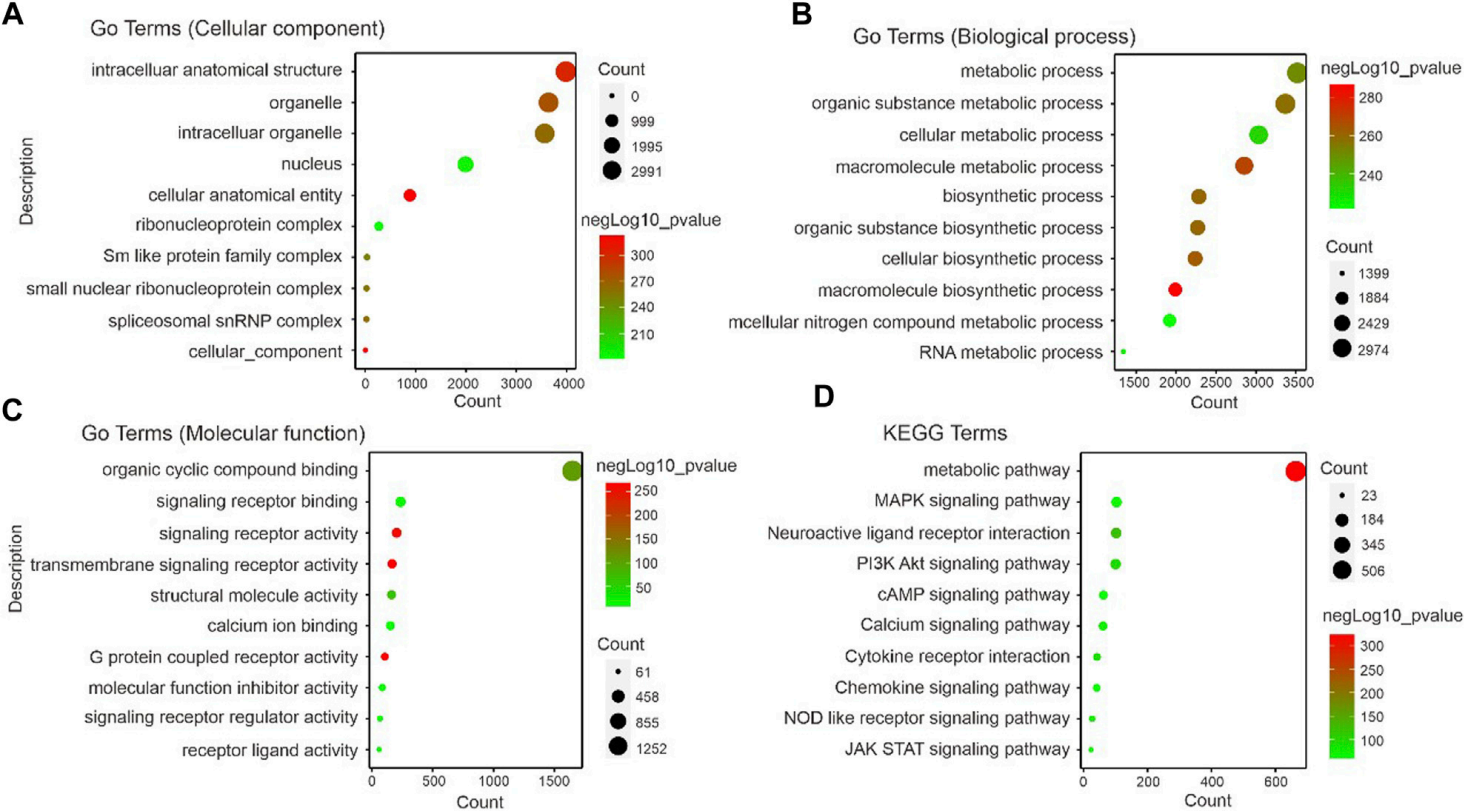
Identification of correlated signaling in DEX-induced analgesic effects **(A–C)**. GO displays the cellular component, biological process and molecular function related signal pathways. Importantly, the results in **(B)** show that a large number of metabolism-related signaling pathways are altered. **(D)**. The foot withdrawal time threshold is associated with the brown module. KEGG displays the enrichment results of signal pathways, indicating the pathways enriched by genes contained in the modules most relevant to PWL analyzed by WGCNA. The size of the dots represents the number of genes enriched to the relevant signal pathways, and the color represents the -log10*p*-value.

A significant correlation was found between gene module and alpha-2 acetylcholine receptor agonism-induced analgesic effects in neuropathic rats (correlation coefficient ranging from 0.71 to 0.54). The functional enrichment analysis of GO and KEGG showed that thousands of signal pathways were obtained, among which metabolism-related pathways had significant changes (Figure 5A–D). Biological process (BP) was mainly associated with metabolism, cellular metabolism, biosynthesis, nitrogen biosynthesis and RNA metabolism (Figure 5B). In molecular function (MF) analysis, signalling receptor binding, transmembrane signalling receptor activity, calcium ion binding and GPCR activity were remarkably enriched (Figure 5C). In addition, KEGG were related to metabolic pathways, MAPK signalling pathways, neuroactive ligand receptor interaction, cAMP signalling pathway, calcium signalling pathway and Jak stat signalling (Figure 5D). These data proved that analgesic effects induced by alpha-2 acetylcholine receptor agonism targeted metabolic capacity and neural plasticity in neuropathic pain.

### Activation of alpha-2 acetylcholine receptors significantly enhances blood supply at the site of spinal cord injury

**FIGURE 6:**
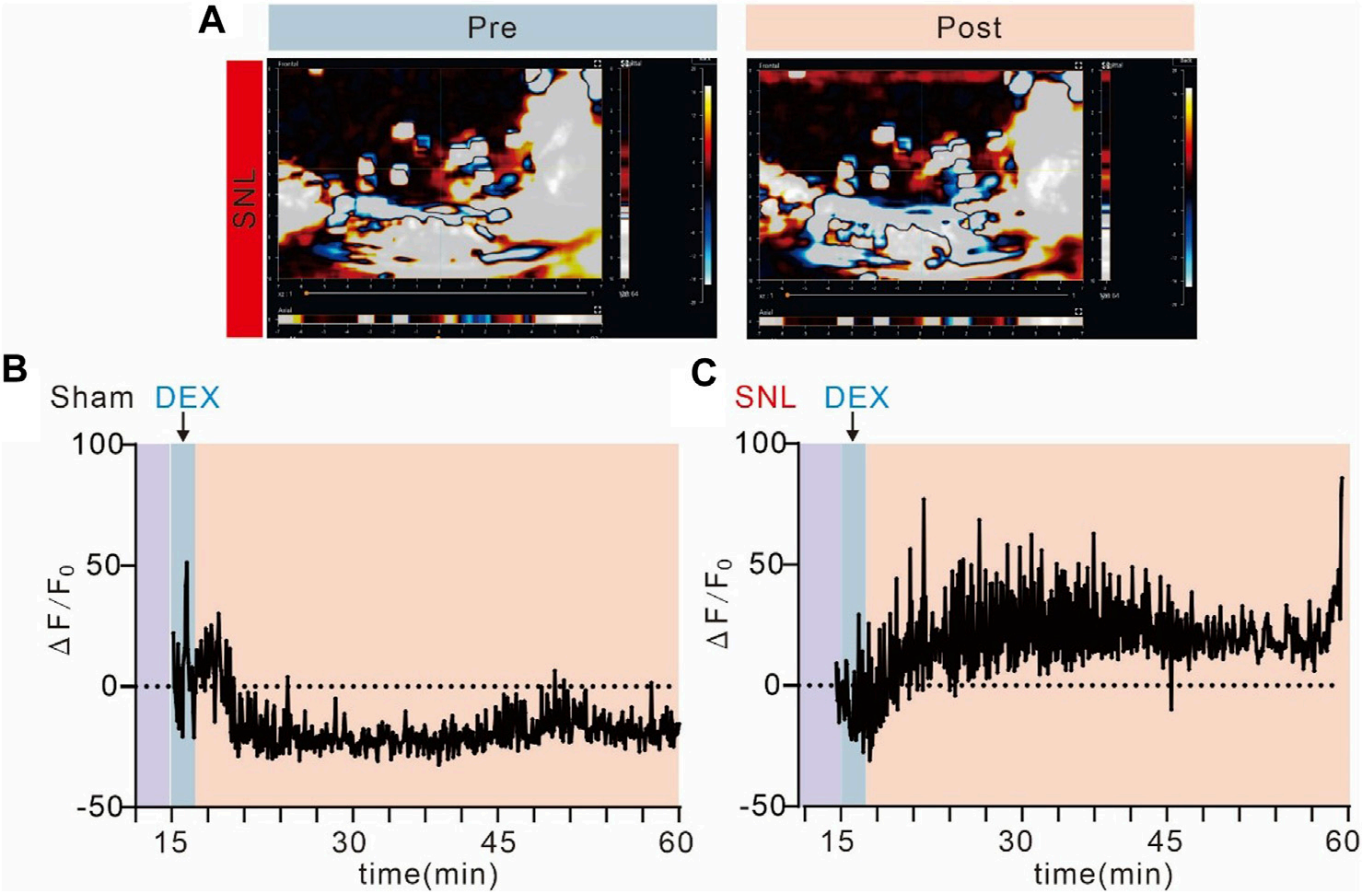
Alpha-2 acetylcholine receptors promote blood supply to spinal cord injuries **(A)**. Ultrasound imaging of spinal cord blood flow in SNL chronic pain mice before and after administration of DEX. **(B, C)**. Spinal cord blood flow in SNL chronic pain mice was significantly enhanced after DEX administration, but not in the sham group.

Chronic pain causes metabolic abnormalities, and the activation of alpha-2 acetylcholine receptors can significantly alleviate pain behaviour. The vascular system should respond to synapse demand. To identify the mechanism of alpha-2 acetylcholine receptors acting on pain sensations, we detected blood flow at the site of spinal cord injury by ultrasonic blood flow imaging (Figure 6A, B). DEX significantly improved blood supply at the site of pain and promoted pain recovery, but it did not affect normal mice. The ΔF value of blood flow in the SNL group was significantly increased after DEX administration (Figure 6C-D). Hence, alpha-2 acetylcholine receptors can enhance blood flow, increase energy supply and promote pain recovery.

### Alpha-2 acetylcholine receptor agonist DEX can upregulate key metabolism-related genes

**FIGURE 7:**
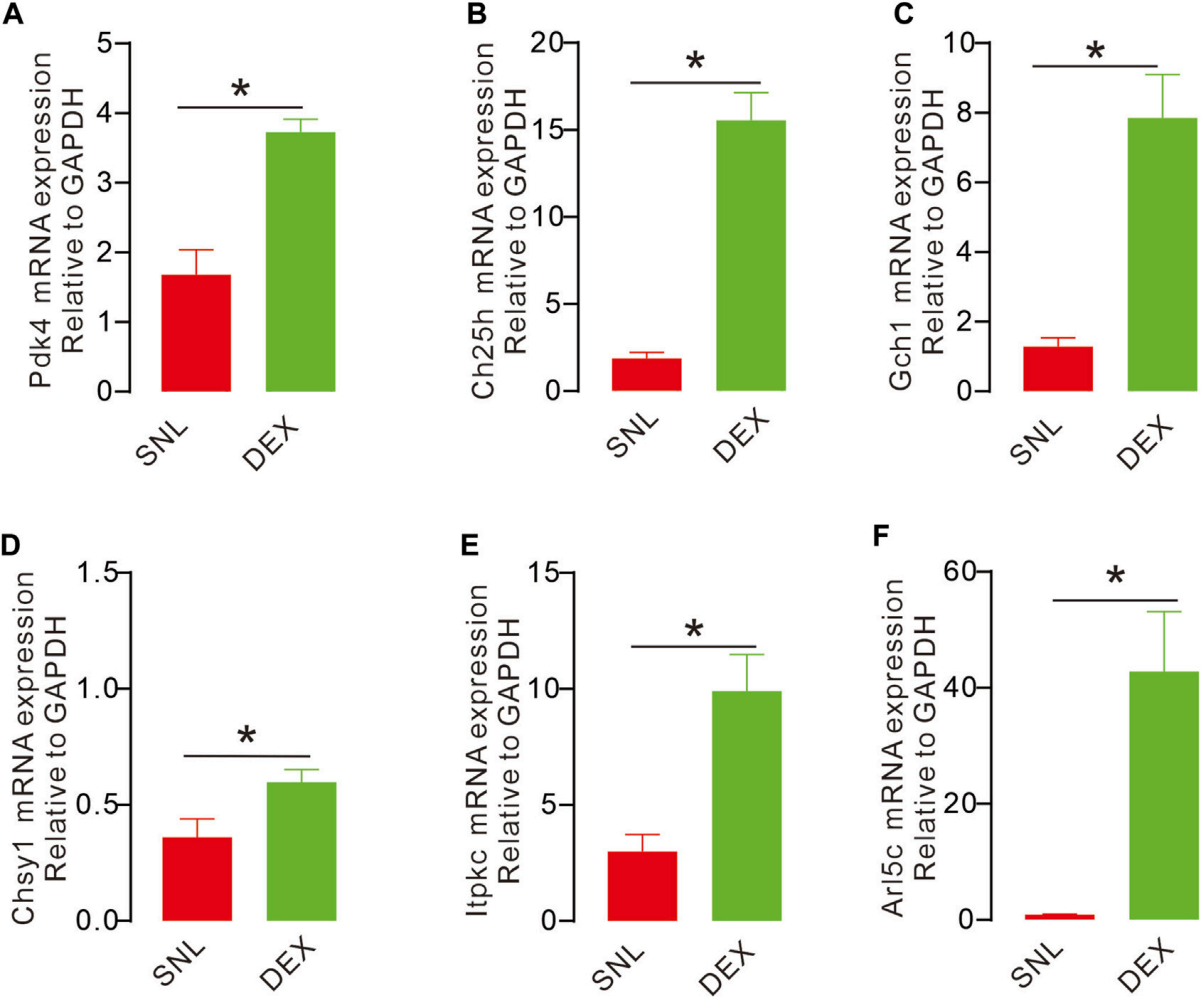
DEX causes upregulation of key metabolism-related genes **(A, C–F)**. The expression of key genes in different groups with QPCR. The expression of Arl5c, Ch25h, Itpkc, Pdk4, Gch1 were significantly increased after injection with DEX, compared with the sham operation group and SNL group, the expression was significant. **(D)**. Compared with the sham operation group, the expression of Chsy1 was significantly decreased. (Pdk4, (t_14_ = 5.068, *p* = 0.0002, *p < 0.05; Ch25h, t_14_ = 8.366, *p* < 0.0001, *p < 0.051; Gch1, t_14_ = 5.156, *p* = 0.0001, *p < 0.05; Chsy1, t_14_ = 2.473, *p* = 0.0268, *p < 0.05; Itpkc, t_14_ = 3.975, *p* = 0.0014, *p < 0.05; Arl5c, t_14_ = 4.051, *p* = 0.0012, *p < 0.05).

After WGCNAs, enrichment gene analysis and ultrasonic imaging verification, we detected the expression of key genes activated by DEX through QPCR because these genes may guide blood and metabolic supply. DEX significantly increased the expression of key genes, such as pyruvate dehydrogenase kinase 4 (Pdk4), cholesterol 25-hydroxylase (Ch25h), GTP cyclohydrolase 1 (Gch1), ADP ribosylation factor-like GTPase 5C (Arl5c), inositol–trisphosphate 3-kinase C (Itpkc) and chondroitin sulphate synthase 1 (Ghsy1) (Figures 7A–F). Our data demonstrated that these genes were remarkably affected in mediating analgesic effects on pain.

### Changes in neuronal plasticity occur at the injury site in rats with chronic pain

**FIGURE 8:**
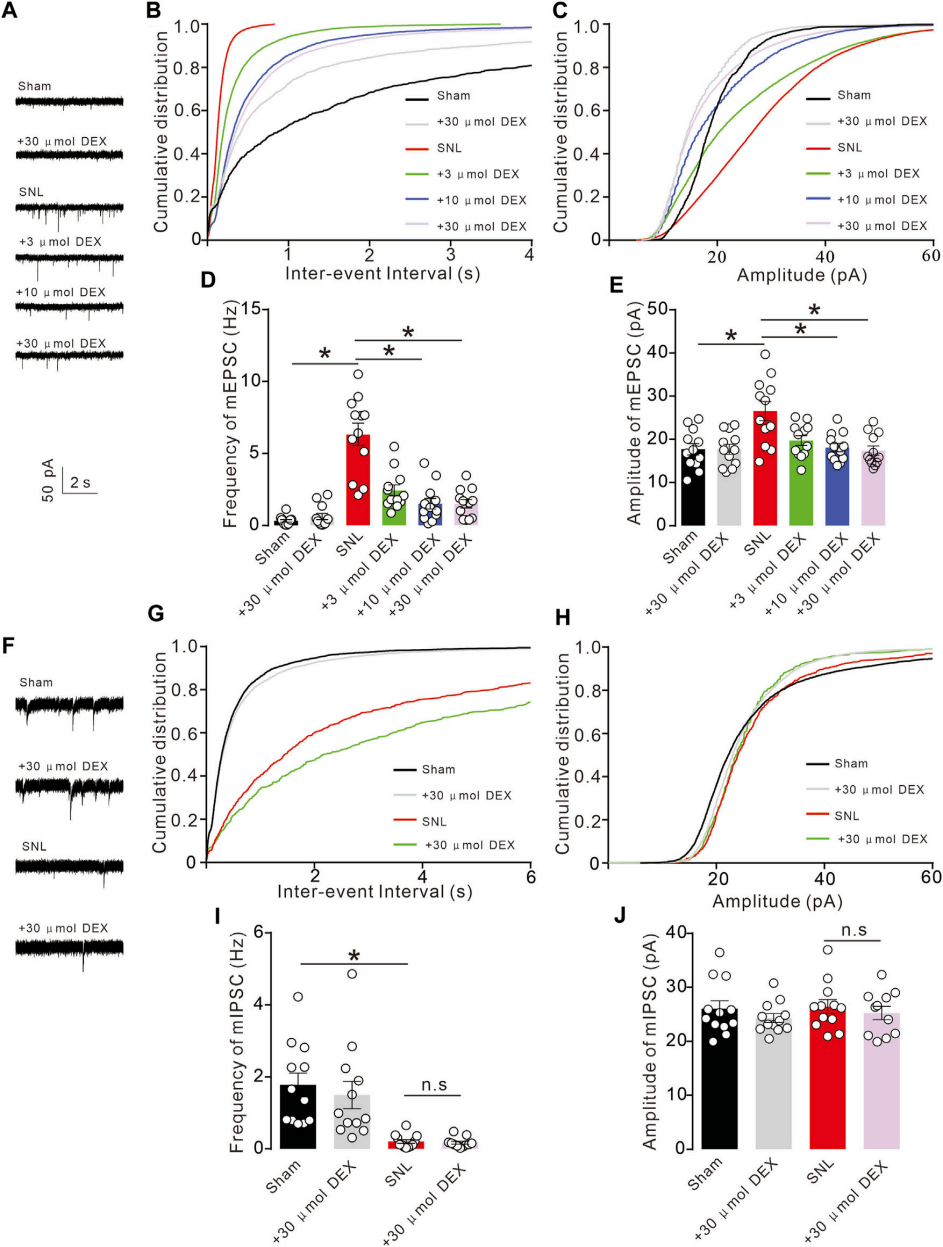
Chronic pain causes abnormal synaptic transmission of spinal cord neurons **(A)**. Chronic SNL pain results in abnormal increase of mEPSC in spinal dorsal horn neurons. **(B–E)**. The increase in mEPSC frequency and amplitude induced by SNL was significantly inhibited by DEX administration (Frequency: F _(5, 66)_ = 27.94, *p* < 0.0001, Amplitude: F _(5, 66)_ = 6.698, *p* < 0.0001, *p < 0.05, two-way ANOVA followed by Sidak’s post-tests). **(F)**. SNL chronic pain can inhibit mIPSC, but giving DEX does not reverse it. **(G–J)**. SNL chronic pain can inhibit mIPSC frequency, but giving DEX does not improve it (Frequency: F _(3, 43)_ = 10.63, *p* < 0.0001, Amplitude: F _(3, 43)_ = 0.6052, *p* = 0.6152, *p < 0.05, two-way ANOVA followed by Sidak’s post-tests).

Chronic pain often causes changes in neuroplasticity. This work aimed to explore changes in electrophysiological indicators of neurons at the injured site of spinal nerve ligation rats and determine if alpha-2 acetylcholine receptors can rescue such changes. By whole-cell patch clamp technique, we recorded the activity of spinal dorsal horn neurons in the sham and SNL groups. By analysing the micro-excitatory postsynaptic current mEPSC and micro-inhibitory postsynaptic current mIPSC, we found that pain caused by spinal nerve ligation significantly increased the frequency and amplitude of mEPSCs of spinal dorsal horn neurons (Figure 8A). These results suggest that pain can induce abnormal enhancement of excitatory synaptic transmission in neurons, and the use of α2-acetylcholine receptor agonist DEX can effectively inhibit the abnormal enhancement (Figures 8B–E). Chronic spinal nerve ligation can also cause abnormal reduction of IPSC, but DEX does not improve it (Figures 8F–J). Collectively, these data demonstrated that DEX produced analgesic effects by alleviating glutamatergic transmission by regulating metabolic demand and vascular response in neuropathic pain.

## Discussion

nAChR is a ligand-gated ion channel formed by the pentameric arrangement of alpha and beta subunits (Mineur et al., 2023). The extracellular domain contains many binding sites of ligands that alter receptors through allosteric mechanisms (Rosenberg et al., 2002). Functional studies have shown that nAChRs help to control resting membrane potential, regulate synaptic transmission and mediate rapid excitatory transmission. The dysfunction of nAChR has been linked to several human diseases, such as schizophrenia, Alzheimer’s and Parkinson’s disease (Spasova et al., 2022). In the present study, the expression of the α2-acetylcholine receptor at the injury site was significantly enhanced in the model of pain induced by nerve injury. Spinal nerve ligation is the most classic chronic pain model, which can cause nerve damage at the lesion site and lead to severe pain reactions. Therefore, SNL was chosen as the pain model in our study. We also observed a significant increase in presynaptic and postsynaptic mRNA expression. The neuropathic pain is mainly related to the imbalance of mitochondrial metabolism and the change in synaptic plasticity. Evidence suggests that α2-acetylcholine receptor agonists are closely related to mitochondrial homeostasis, and DEX significantly enhances the level of blood perfusion in the spinal cord, thereby confirming its regulatory function on energy metabolites required by mitochondria. In whole-cell perfusion experiments, we observed that DEX inhibited spinal cord glutaminergic neurotransmission in a dose-dependent manner, which may be related to its function in regulating mitochondrial metabolism. In summary, our study observed a correlation between DEX and pain at the global and neuronal levels.

Mitochondria are the only site of ATP synthesis, and it is necessary to guide nearby blood vessels to dilate through AMPK and NO to increase the blood supply at the mitochondrial site of local synaptic sites (Gou et al., 2023). However, the contents of neurons at the site of nerve injury will diffuse out of the cell, in which ATP is an important inflammatory mediator. ATP binds to receptors such as P2Y12, resulting in spinal cord glutaminergic neurotransmission. SNL significantly increased P2Y12 receptor levels, and injection of a P2Y12 antagonist (MRS2395 or clopidogrel) attenuated microglial activation; partial sciatic nerve ligation (PSNL) induced excessive micro-excitatory postsynaptic current (mEPSC), and this effect was significantly attenuated in P2Y12-knocked out mice (Yu et al., 2019). Abnormal levels of ATP in blood vessels are associated with a variety of diseases, such as inflammation, hypoxia and atherosclerosis. The heterogeneous distribution of ATP in the network, the heterogeneity between different concentrations and the same level of blood vessels play an important role in performing different functions (Gou et al., 2023). Astrocyte expression is closely related to ATP in the regulation of neuronal activity. Neurotransmitter receptors, including the P2 receptor, can release ATP in response to various stimuli and cause physiological reactions in response to extracellular ATP (Gu and Wiley, 2006). This process has reciprocal feedback with calcium ions and participates in neural activity. In addition, astrocytes direct the communication between neurons and blood vessels through their connecting blood vessels. In conclusion, ATP-mediated dynamic communication among neurons, astrocytes and blood vessels is a key molecule for information processing or integration in the central nervous system. Thus, theoretically, we observed the dynamic relationship between DEX and blood flow.

Synaptic transmission is a dynamic energy-consuming process and includes biological metabolism, which is based on ATP activity. Mitochondrial damage causes many diseases. In spinal cord injury, mitochondrial imbalance is the main feature that leads to irreversible body disability (Kang et al., 2020). ROS is a product of mitochondrial oxidative metabolism and is involved in maintaining REDOX balance. In physiological states, ROS production and elimination balance each other. However, in pathological states such as nerve injury, the contents of neurons overflow, leading to mitochondrial breakage and ROS accumulation in tissue parts, resulting in mitochondrial damage, further expanding the chaotic mitochondrial oxidative damage and developing into serious mitochondria-mediated nervous system diseases (Smirnova et al., 2001). Therefore, mitochondrial homeostasis should be regulated and neural activity should be inhibited by regulating the α2-acetylcholine receptor. Neuronal energy metabolism is a complex life process designed for basic pathways of glucose metabolism: glycolysis, gluconeogenesis, glycogenesis, lipid, cholesterol, and glycogenolysis, which may engage in synaptic transmission. Our results revealed that cholesterol hydroxylase promotes cholesterol metabolism in extrahepatic tissues by converting cellular cholesterol to circulating oxysterols, thereby regulating various biological processes, especially in pain states; the upregulation of cholesterol hydroxylase (CH25H) remarkably increased the production of oxysterol metabolites and guide neuronal metabolic homeostasis (Choi et al., 2019). PDK4 is a repressor of the conversion of pyruvate into acetyl-CoA by the PHD complex in regulating ATP homeostasis during pathologic states. We demonstrated that PDK4 was upregulated following α2-acetylcholine agonism and promoted the ATP supply.

Therefore, in our study, we further support transcriptomic studies that demonstrated through FUS and whole-cell records that mitochondrial metabolic homeostasis is the main cause of α2-acetylcholine receptor analgesia. Damage to the binding of DEX, its agonist, leads to upregulated receptors that promote the communication between blood vessels and local synapses, thereby regulating mitochondrial ATP production and playing an analgesic role.

## Conclusion

**FIGURE 9:**
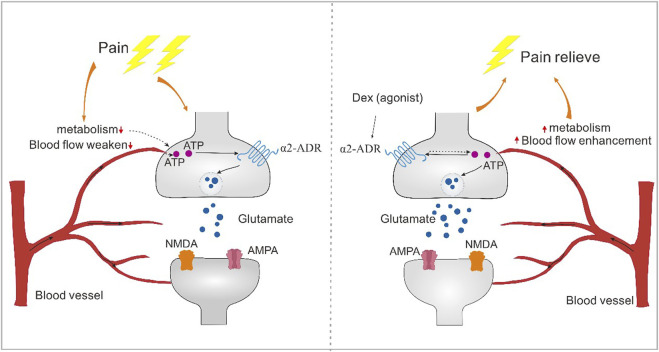
The alpha2-acetylcholine receptor regulates pain through energy metabolism. Blood vessels have the effect of responding to tissue metabolism. Blood flow at the pain site is weakened, resulting in abnormal energy supply to neurons, resulting in abnormal synaptic transmission. α2-acetylcholine receptors can enhance blood flow, increase the regulation of neuronal activity, reverse abnormal changes in synaptic plasticity, and thus regulate the occurrence of pain.

Blood vessels respond to tissue metabolism. Blood flow at the pain site is weakened, resulting in abnormal energy supply to neurons and abnormal synaptic transmission. α2-Acetylcholine receptors can enhance blood flow, increase the regulation of neuronal activity, reverse abnormal changes in synaptic plasticity and regulate the occurrence of pain (Figure 9).

## Data Availability

The original contributions presented in the study are publicly available. This data can be found here: https://www.ncbi.nlm.nih.gov/bioproject/1082875.
